# Mitochondrial Genome Sequencing and Phylogenetic Analysis of *Myotis pequinius* and *M. chinensis*

**DOI:** 10.1002/ece3.74219

**Published:** 2026-08-20

**Authors:** Xufan Wang, Xiangxiang Cao, Haiyan Guan, Huigen Feng, Sen Liu

**Affiliations:** ^1^ North Henan Medical University Xinxiang Xinxiang Henan China; ^2^ College of Life Sciences Henan Normal University Xinxiang Henan China; ^3^ The Observation and Research Field Station of Taihang Mountain Forest Ecosystems of Henan Province Xinxiang Henan China

**Keywords:** *M. chinensis*, Mitogenome, *Myotis pequinius*, phylogenetic analysis

## Abstract

This study first sequenced and analyzed the mitochondrial genomes of *Myotis pequinius* and *M. chinensis* . The results showed that the mitochondrial genome of *M. pequinius* and *M. chinensis* were 17,395 bp and 17,014 bp, respectively. Both genomes presented a typical mammalian mitochondrial composition, including 13 protein‐coding genes, 22 tRNA genes, two rRNA genes and one D‐loop region, with an obvious AT preference. The phylogenetic tree based on 13 protein‐coding genes showed that *M. pequinius* and *M. chinensis* were genetically close to each other, and they were clustered with *M. bombinus* , *M. nattereri* , *M. myotis* , and *M. blythii* into a high‐support independent branch. This study fills the mitogenomic data gap of the two Chinese bat species, provides basic molecular resources for resolving the taxonomic controversies of the genus *Myotis*, and offers valuable genetic references for the biodiversity conservation and population genetic research of local bat populations.

## Graphical abstract

This study first determined the complete mitochondrial genomes of *Myotis pequinius* (17,395 bp) and *M. chinensis* (17,014 bp), which both contain 13 protein‐coding genes, 22 tRNAs, 2 rRNAs, and a D‐loop, exhibiting an AT‐rich base composition. Phylogenetic analysis based on the 13 protein‐coding genes revealed that the two species are closely related and cluster with *M. bombinus* , *M. nattereri* , *M. myotis* , and *M. blythii* as a highly supported independent branch, clarifying their evolutionary position within the genus *Myotis*.

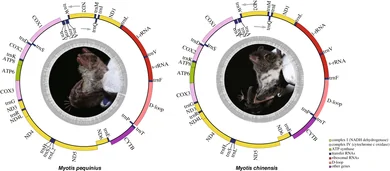

## 1 Introduction

With the rapid development of modern molecular biology, the analysis of species evolution based on molecular data has become a hot topic in biological research. The mitochondrial genome has become an ideal tool for studying the phylogenetic relationships of mammals because of its maternal inheritance, rapid evolutionary rate, and relatively simple structure (Tzameli 2012). Among them, the coevolutionary patterns of 13 protein‐coding genes can provide high‐resolution information for phylogenetic reconstruction and are widely used in studies of species origin, phylogenetic relationships, and genetic diversity.

*Myotis*, belonging to the order Chiroptera and family Vespertilionidae, is one of the most widely distributed and species‐diverse terrestrial vertebrate groups globally, with an extremely diverse ecological niche. Accurate species identification is a crucial foundation for understanding biodiversity and conducting evolutionary studies (Sites and Marshall 2003). However, early taxonomic classification primarily relied on morphological characteristics, dividing the genus into three to seven subgenera (Liu et al. 2023); yet the similarity in morphological features may stem from convergent adaptation to similar ecological niches, making it difficult to accurately reflect phylogenetic relationships (Ghazali et al. 2017; Morales et al. 2019). In recent years, Guan et al. systematically elucidated the phylogenetic relationships within Chiroptera based on 219 mitochondrial genomes (covering 187 bat species, including 54 newly sequenced) and 200 nuclear gene orthologous sequences (Guan et al. 2025). The mitochondrial genome demonstrates distinct advantages in providing high‐resolution phylogenetic signals, enabling more effective support for phylogenetic reconstruction and species relationship analysis (Monzel et al. 2023). It is important to acknowledge that in bats, and particularly within the genus *Myotis*, mitochondrial DNA should be interpreted with caution when used alone for phylogenetic and taxonomic inference. Well‐documented phenomena such as mitochondrial introgression, incomplete lineage sorting, and mito‐nuclear discordance have been extensively reported in this genus (Puechmaille 2026; Çoraman et al. 2020). Platt et al. (2018) sequenced full mitochondrial genomes and 3648 ultraconserved elements (UCEs) from 37 representative *Myotis* species and found that all 294 phylogenies generated from nuclear UCE data differed significantly from those inferred using mitochondrial genomes, with approximately half of all UCE loci conflicting with the estimated species tree. These findings suggest that the mitochondrial genome may be as likely to reflect the true species tree as any single nuclear locus chosen at random, and that phenomena such as lineage sorting, reticulation, and introgression have likely influenced the genomes of *Myotis* (Platt et al. 2018). Therefore, while mitochondrial genomes provide valuable data for generating phylogenetic hypotheses, their limitations must be explicitly recognized. However, the NCBI database currently contains only 36 mitochondrial genomes of the genus *Myotis*, and the information coverage remains insufficient, severely restricting the in‐depth development of phylogenetic studies on this genus, especially in China.

As a species endemic to China, *M. pequinius* is currently known to be distributed only in Beijing, Hebei, Shaanxi, Sichuan, Henan, Anhui, Jiangsu, and Shandong, exhibiting pronounced geographical restriction and habitat specificity. Although *M. chinensis* is widely distributed across most of southern China, mitochondrial genomic data for these two species remain entirely lacking at present. This deficiency leaves the phylogenetic framework of the genus *Myotis* in China without solid data support, resulting in ambiguous taxonomic boundaries and underscoring an urgent need for systematic molecular investigations.

This study sequenced and annotated the mitochondrial genomes of both *M. pequinius* and *M. chinensis* , systematically analyzing their structural characteristics and codon usage preferences. On this basis, combining the existing mitochondrial genome data of *Myotis* from the NCBI database, a maximum likelihood method was used to construct a phylogenetic tree, aiming to preliminarily explore the mitochondrial lineage relationships of the two species and provide foundational mitogenomic data for future integrative taxonomic studies. It is important to emphasize that the phylogenetic results presented here represent a mitochondrial gene tree, which does not necessarily equate to the species tree; validation with nuclear markers is required for robust taxonomic conclusions. The research findings not only provide valuable mitochondrial genomic resources for future taxonomic revision of the genus *Myotis* in China, but also offer important scientific references for the biodiversity conservation, systematic evolution studies, and genetic resource management of species within this genus.

## 2 Materials and Methods

### 2.1 Sampling and Storage

*M. pequinius* was collected in Lingqiu County, Shanxi Province, China, and *M. chinensis* was collected in Shangcheng County, Henan Province, China. All samples were adult male bats. The tissue of 2 mm diameter was taken and stored in 95% ethanol, and then stored at −20°C until DNA was extracted. The sampling procedure was approved by the Academic Ethics and Ethics Committee of Henan Normal University (HNSD‐2025BS‐1005).

### 2.2 DNA Extraction and Sequencing

Genomic DNA was extracted from the pterodactyl membrane using the kit (DE712‐50) provided by Beijing Jinsha Biotechnology Co. Ltd. A total of 200 ng of genomic DNA was extracted and processed into a DNA library using the standard protocol of the VAHTS Universal Plus DNA Library Prep Kit for MGI (CAT#NDM617‐02, Vazyme). The library was then subjected to high‐throughput sequencing on the DNBSEQ‐T7 platform. The downloaded data were filtered and deduplicated using Fastp (Chen et al. 2018) (v0.23.2), followed by quality assessment with FastQC (v0.11.9). The mitochondrial genome was assembled using the GetOrganelle software (v1.7.6.1), followed by genome annotation with MitoZ (v3.6). After quality control, the sequencing data were aligned to the final mitochondrial genome sequence using bwa (v0.7.12‐r1039). Table S1 presents a summary of sequencing data and assembly coverage for the mitochondrial genomes of the two species. The annotated mitochondrial genome sequence was submitted to the GenBase at the National Bioinformation Center of China (https://ngdc.cncb.ac.cn/gsa). The newly assembled mitochondrial genome sequences of *M. pequinius* and *M. chinensis* have been deposited in GenBase, the repository of the National Bioinformation Center of China (https://ngdc.cncb.ac.cn/genbase/), under accession numbers C_AA119463.1 and C_AA159671.1, respectively.

### 2.3 Sequence Analysis

The sequence operation toolbox JavaScrip (http://www.detaibio.com/sms2/codon_plot.html) was used to calculate the base composition ratios of mitochondrial genome, protein‐coding genes, trna (transfer RNA), rRNA (ribosomal RNA), and the D‐loop (displacement loop), as well as the AT/GC ratios, AT bias values, and GC bias values. The relative synonymous codon usage (RSCU) of each protein‐coding gene was calculated using DnaSP v5 software (Librado and Rozas 2009) to evaluate codon usage preferences. The nucleotide substitution rate (*Ka/Ks*) of nonsynonymous mutation frequency (*Ka*) to synonymous mutation frequency (*Ks*) for each coding gene was calculated to assess selection pressure. *Ka/Ks* was used to determine the gene differentiation level: when *Ka/Ks* > 1, it indicates positive selection; when *Ka/Ks* < 1, it indicates purifying selection; and when *Ka/Ks* = 1, it indicates neutral evolution (Wang et al. 2009). The *Ka/Ks* values for each gene were calculated by pairwise comparisons among different *Myotis* species. The secondary structures of mitochondrial tRNAs in *M. chinensis* and *M. pequinius* were predicted using the online tool MITOS (http://mitos.bioinf.uni‐leipzig.de/index.py) and visualized on the ViennaRNA website (http://rna.tbi.univie.ac.at/forna/). Structural characteristics of the D‐loop region were obtained by referencing other reported D‐loop structures in bats (Sun et al. 2009; Rahman et al. 2019; Barrera et al. 2023; Martínez‐ Cárdenas et al. 2024).

### 2.4 Phylogenetic Analysis

Maximum likelihood (ML) was used to construct the phylogenetic tree. Mitochondrial genomes of 34 *Myotis* species were downloaded from the NCBI database, with the mitochondrial genomes of *Murina leucogaster* and *Kerivoula minuta* serving as the outgroup (Table 1).

**TABLE 1:** Mitochondrial genome information for the 34 *Myotis* species and two outgroup species involved in this study.

Species	Accession number	Species	Accession number
*Myotis atacamensis*	NC_036324	*Myotis macrodactylus*	NC_022694
*Myotis aurascens*	NC_060697	*Myotis martiniquensis*	NC_036328
*Myotis bechsteinii*	NC_034227	*Myotis muricola*	NC_029422
*Myotis blythii*	MT588108	*Myotis myotis*	NC_029346
*Myotis bombinus*	MT985383	*Myotis nattereri*	MN122818
*Myotis brandtii*	NC_025308	*Myotis nigricans*	NC_036318
*Myotis dasycneme*	MN122855	*Myotis oxyotus*	NC_036316
*Myotis davidii*	NC_025568	*Myotis petax*	NC_056773
*Myotis dominicensis*	NC_036312	*Myotis pilosus*	MN245054
*Myotis evotis*	NC_036313	*Myotis planiceps*	OR752434
*Myotis findleyi*	OR752436	*Myotis riparius*	NC_036317
*Myotis formosus*	NC_015828	*Myotis ruber*	NC_036315
*Myotis frater*	NC_041638	*Myotis septentrionalis*	NC_049871
*Myotis horsfieldii*	NC_036325	*Myotis thysanodes*	NC_036323
*Myotis ikonnikovi*	NC_022698	*Myotis volans*	NC_036326
*Myotis keaysi*	NC_036314	*Myotis yumanensis*	NC_036319
*Myotis leibii*	NC_036321	*Kerivoula minuta*	NC_061569
*Myotis lucifugus*	NC_029849	*Murina leucogaster*	NC_025949

The 13 protein‐coding gene sequences of each individual were tandemly sequenced and aligned using Geneious Pro 4.8.2 software (Biomatters Ltd., Auckland, New Zealand). The best nucleotide substitution model was calculated by MEGA 11.0 software as GTR + G + *I. maximum* Likelihood (ML) phylogenetic analysis of the concatenated sequences was performed using RAxML‐HPC BlackBox v8.1.24 software on the CIPRES Science Gateway website (https://www.phylo.org/), with 1000 bootstrap replicates. The generated ML phylogenetic trees were visualized using FigTree v1.4.4 software (http://tree.bio.ed.ac.uk/software/figtree/).

## 3 Results

### 3.1 Structure and Base Composition of the Mitochondrial Genome

**FIGURE 1:**
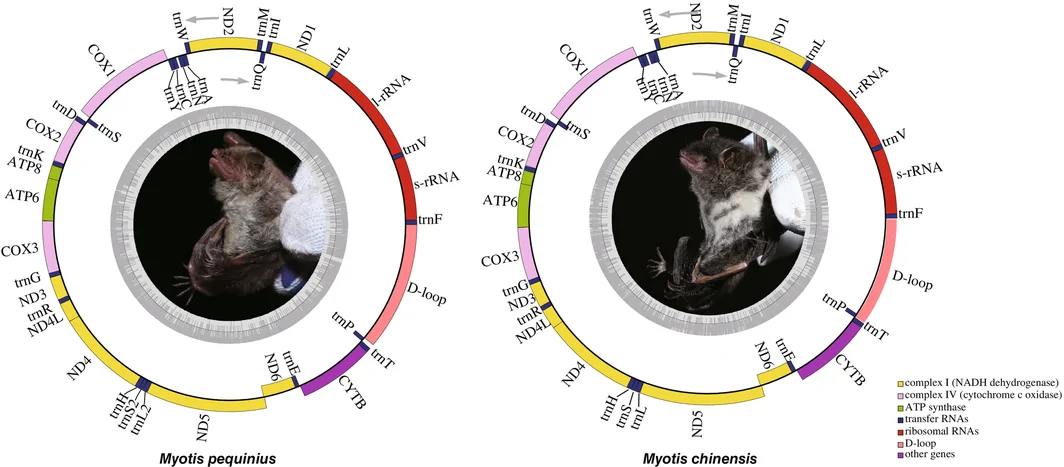
Mitochondrial genome map of *Myotis pequinius* and *M. chinensis* . These 38 genes are encoded on two DNA strands. Protein‐coding genes and rRNA genes are denoted by standard abbreviations. For tRNAs, each letter corresponds to the specific amino acid they carry, with two types of leucine tRNAs and two types of serine tRNAs distinguished numerically.

The mitochondrial genome of *M. chinensis* (17,014 bp) and *M. pequinius* (17,395 bp) are both typical double‐stranded circular molecules. The mitochondrial genome of these species exhibit structural and gene arrangement consistency with reported bat mitochondrial genomes, comprising 13 PCGs (Protein‐Coding Genes), 22 tRNAs, 2 rRNAs, and a D‐loop (Figure 1).

The eight tRNAs (*trnS2*, *trnE*, *trnP*, *trnQ*, *trnA*, *trnN*, *trnC*, *trnY*) and *ND6* of *M. pequinius* and *M. chinensis* are encoded by L chain, while the remaining genes are located on H chain (Table S2). The longest gene sequence in the genomes of the two species of *Myotis* is *ND5*, which is 1821 bp. The genome exhibits multiple gene overlaps, with the longest overlapping region located between *ATP8* and *ATP6* (−43 bp), indicating a highly compact genomic structure. The intergenic regions range from 1 bp to 32 bp, with the longest interval between *trnN* and *trnC* (32 bp).

The s‐rRNA and l‐rRNA lengths of two bat species are different: in the genome of *M. pequinius* , the s‐rRNA and l‐rRNA lengths are 970 bp and 1568 bp respectively, while in the mitochondrial genome of *M. chinensis* , the s‐rRNA and l‐rRNA lengths are 975 bp and 1562 bp respectively.

### 3.2 Nucleotide Composition

The analysis of the base composition in each functional region of the mitochondrial genome of *M. pequinius* revealed that all regions maintained the AT bias characteristic of the total genome, with certain differences in base bias among different regions (Table 2). The data reveal that the PCGs region exhibits a 64.98% A + T content, marginally higher than the whole genome, with an AT‐Skew of −0.01—nearly zero—indicating the most balanced base usage where A and T are nearly equal. The tRNA region, with 65.80% A + T content and an AT‐Skew of 0.01, ranks second in base equilibrium after PCGs. Its GC‐Skew of 0.09 was the only region with a weak positive GC‐Skew, showing slightly higher G content than C, which deviates slightly from the GC bias patterns observed in the whole genome and other regions. The A + T content in the rRNA region (including 12S rRNA and 16S rRNA) was 64.30%, with 16S rRNA exhibiting a higher A + T content (66.14%) than 12S rRNA (61.34%). Both regions showed positive AT‐Skew values, where 12S rRNA demonstrated a stronger A‐base preference (0.24) compared to 16S rRNA (0.20). The D‐loop region had the lowest A + T content (60.43%) among all regions, yet its GC‐Skew value (−0.24) maintained the overall genomic bias of higher C content than G.

**TABLE 2:** Structural components and skew of the mitochondrial genomes of *Myotis pequinius* and *M. chinensis* .

region		A	T	G	C	G + C%	A + T%	AT‐Skew	GC‐Skew
Mitogenome	*M. pequinius*	34.18	30.25	12.82	22.75	35.57	64.43	0.06	−0.28
	*M. chinensis*	34.47	30.76	12.59	22.18	34.77	65.23	0.06	−0.28
PCGs	*M. pequinius*	32.05	32.93	12.30	22.72	35.02	64.98	−0.01	−0.30
	*M. chinensis*	33.44	31.87	10.76	23.93	34.69	65.31	0.02	−0.38
tRNA	*M. pequinius*	33.20	32.60	18.56	15.64	34.20	65.80	0.01	0.09
	*M. chinensis*	38.24	25.00	16.18	20.59	36.77	63.24	0.21	−0.12
rRNA	*M. pequinius*	39.00	25.30	16.47	19.23	35.70	64.30	0.21	−0.08
	*M. chinensis*	37.33	24.10	17.64	20.92	38.56	61.43	0.22	−0.09
12S rRNA	*M. pequinius*	37.94	23.40	17.22	21.44	38.66	61.34	0.24	−0.11
	*M. chinensis*	37.33	24.10	17.64	20.92	38.56	61.43	0.22	−0.09
16S rRNA	*M. pequinius*	39.67	26.47	16.00	17.86	33.86	66.14	0.20	−0.05
	*M. chinensis*	38.99	26.89	16.33	17.80	34.13	65.88	0.18	−0.04
D‐loop	*M. pequinius*	33.00	27.43	15.03	24.54	39.57	60.43	0.09	−0.24
	*M. chinensis*	36.99	30.83	11.37	20.81	32.18	67.82	0.09	−0.29

The base composition of functional regions in the mitochondrial genome of *M. chinensis* generally exhibits AT‐enriched characteristics similar to those of *M. pequinius* , but significant differences in base bias are observed in certain regions (Table 2). The A + T content in the PCGs region of this species is 65.31%, slightly higher than that of *M. pequinius* . Its AT‐Skew is 0.02, showing a weak positive value, indicating that the A content is slightly higher than T, which is contrary to the characteristic of *M. pequinius* where the A content in the PCGs region is slightly lower than T. Moreover, its GC‐Skew (−0.38) is significantly lower than that of *M. pequinius* (−0.30). The tRNA region of *M. chinensis* is a low A + T region (63.24%), which is different from the high A + T region of *M. pequinius* . The GC‐Skew (−0.12) of the tRNA region of *M. chinensis* is negative, which is different from the base bias pattern of the tRNA region of *M. pequinius* . The rRNA region (with sequences identical to 16S rRNA) exhibits an A + T content of 61.43% and an AT skew of 0.22, consistent with the A‐preferential characteristics observed in the rRNA region of *M. pequinius* . The D‐loop region demonstrates the highest G + C content among all regions of this species, reaching 67.82%, with a GC skew of −0.29. This region maintains the C‐to‐G bias, with a higher degree of bias compared to *M. pequinius* .

### 3.3 Protein‐Coding Genes and Codon Usage Patterns

The total length of 13 PCGs in the mitochondrial genomes of *M. pequinius* and *M. chinensis* is 11,440 bp, and the percentage of genes in the total length of mitochondrial genome is 65.77% ( *M. pequinius* ) and 67.23% ( *M. chinensis* ) respectively. Most of the PCGs are separated by one or more tRNAs. Except for *ND6* which is encoded by L chain, all other PCGs are encoded by H chain. *ND5* (1821 bp) is the longest protein‐coding gene, while *ATP8* (204 bp) is the shortest. The size and structure of the 13 PCGs show no significant differences compared to other bat species. In terms of start codons, except for *ND3*, *ND5*, and *ND2* which use ATA, ATA, and ATT as their start codons respectively, the other 10 protein‐coding genes all utilize ATG as their start codon. Termination codons exhibit remarkable diversity: *COX1*, *COX2*, *ATP8*, *ATP6*, *ND4L*, *ND5*, *ND6*, and *ND1* utilize TAA as their termination codon; *COX3*, *ND3*, and *ND2* employ TAG as their termination codon; the ACT termination codon of *ND4* is particularly unique, potentially forming a complete TAA termination codon through post‐transcriptional polyadenylation. In terms of codons, the termination codons of *CYTB* in the mitochondrial genomes of *M. pequinius* and *M. chinensis* are different, which are AGA and TAA respectively.

**FIGURE 2:**
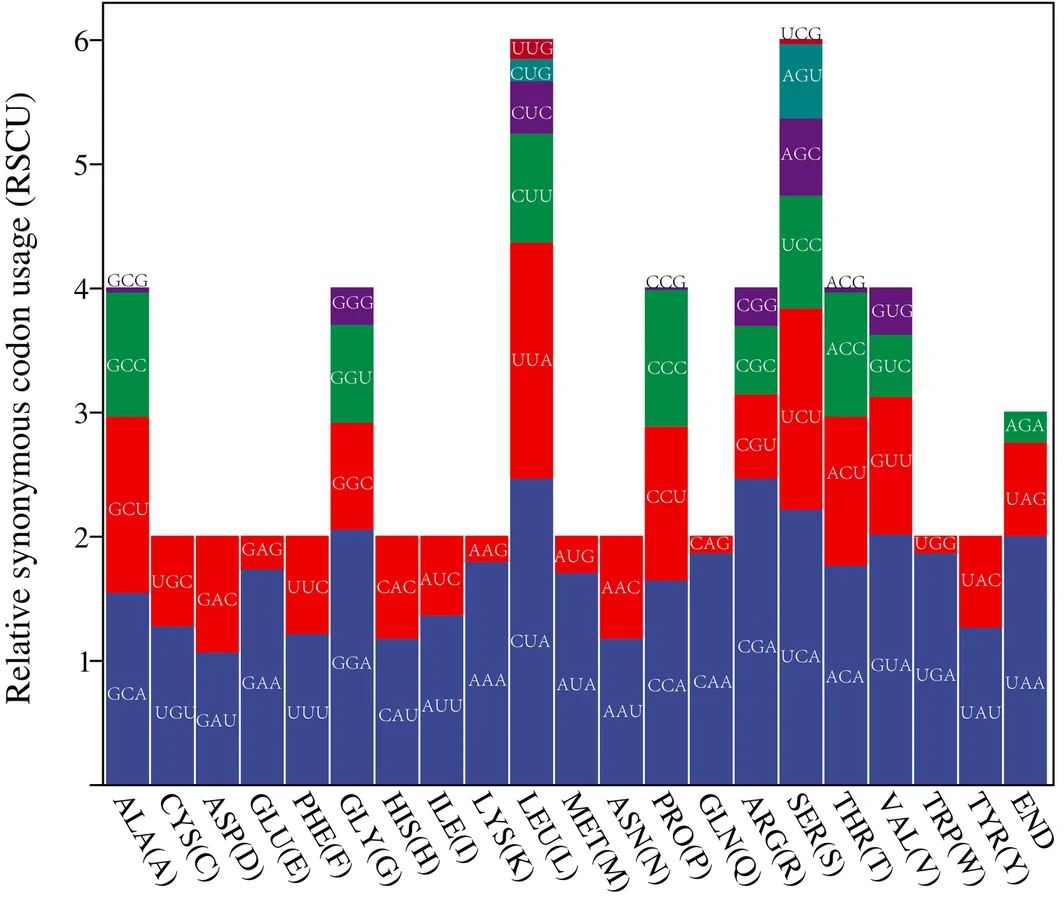
Relative synonymous codon usage (RSCU) of the PCGs of *M. pequinius*.

The 13 protein‐coding genes of both *M. pequinius* and *M. chinensis* use multiple codons to encode each amino acid. Analysis of the codon usage pattern in *M. pequinius* revealed a codon usage bias in their genome (Figure 2): there is a general preference for codons ending with adenine (A) or uracil (U) (Table 3). Among them, the codon GGA is the most preferred codon in this species, with a relative synonymous codon usage (RSCU) as high as 1.96. Several other high‐frequency codons, such as CUA (Leu, RSCU = 1.83), GUA (Val, RSCU = 1.81), CAA (Gln, RSCU = 1.79), and CCU (Pro, RSCU = 1.76), also end with A or U, collectively forming the fundamental codon usage pattern of this species. Meanwhile, some codons are less frequently used, with the GCU codon for Ala (RSCU = 0.06) being the least frequently used among all codons.

**TABLE 3:** Frequency and RSCU values of codon in PCGs of *M. chinensis* and *M. pequinius* .

Amino acid	Codon	M. chinensis		M. pequinius		Amino acid	Codon	M. chinensis		M. pequinius	
		Count	RSCU	Count	RSCU			Count	RSCU	Count	RSCU
Ala(A)	GCA	67	1.51	49	1.45	Pro(P)	CCG	3	0.05	7	0.13
	GCU	57	1.29	52	1.54		CCA	74	1.36	62	1.13
	GCC	45	1.02	32	0.95		CCU	93	1.71	97	1.76
	GCG	8	0.18	2	0.06		CCC	48	0.88	54	0.98
Cys(C)	UGU	36	1.36	40	1.14	Gln(Q)	CAG	7	0.18	8	0.21
	UGC	17	0.64	30	0.86		CAA	70	1.82	67	1.79
Asp(D)	GAU	32	1.31	33	1.14	Arg(R)	CGG	15	0.66	14	0.73
	GAC	17	0.69	25	0.86		CGA	42	1.87	27	1.4
Glu(E)	GAG	7	0.19	11	0.38		CGU	13	0.58	15	0.78
	GAA	65	1.81	47	1.62		CGC	20	0.89	21	1.09
Phe(F)	UUU	131	1.25	143	1.33	Ser(S)	AGU	40	0.75	52	0.88
	UUC	78	0.75	72	0.67		AGC	60	1.12	86	1.46
Gly(G)	GGG	19	0.48	10	0.31		UCG	9	0.17	11	0.19
	GGA	74	1.86	64	1.95		UCA	92	1.73	70	1.19
	GGU	31	0.78	30	0.92		UCU	74	1.39	90	1.53
	GGC	35	0.88	27	0.82		UCC	45	0.84	44	0.75
His(H)	CAU	75	1.2	103	1.27	Thr(T)	ACG	10	0.13	13	0.17
	CAC	50	0.8	59	0.73		ACA	126	1.55	94	1.16
Ile(I)	AUU	232	1.45	213	1.4		ACU	122	1.5	140	1.73
	AUC	88	0.55	92	0.6		ACC	67	0.82	76	0.94
Lys(K)	AAG	10	0.19	14	0.24	Val(V)	GUG	8	0.25	8	0.28
	AAA	96	1.81	105	1.76		GUA	67	2.11	51	1.81
Leu(L)	UUG	23	0.26	19	0.25		GUU	27	0.85	32	1.13
	UUA	161	1.82	115	1.53		GUC	25	0.79	22	0.78
	CUG	23	0.25	31	0.42	Trp(W)	UGG	33	0.58	41	0.73
	CUA	181	2.04	138	1.83		UGA	81	1.42	71	1.27
	CUU	92	1.04	99	1.31	Tyr(Y)	UAU	136	1.37	145	1.35
	CUC	52	0.59	50	0.66		UAC	62	0.63	70	0.65
Met(M)	AUG	38	0.34	48	0.48	End	AGA	26	0.95	41	1.07
	AUA	184	1.66	154	1.52		UAG	11	0.4	9	0.24
Asn(N)	AAU	136	1.29	171	1.29		UAA	44	1.62	55	1.44
	AAC	75	0.71	94	0.71		AGG	28	1.03	48	1.25

**FIGURE 3:**
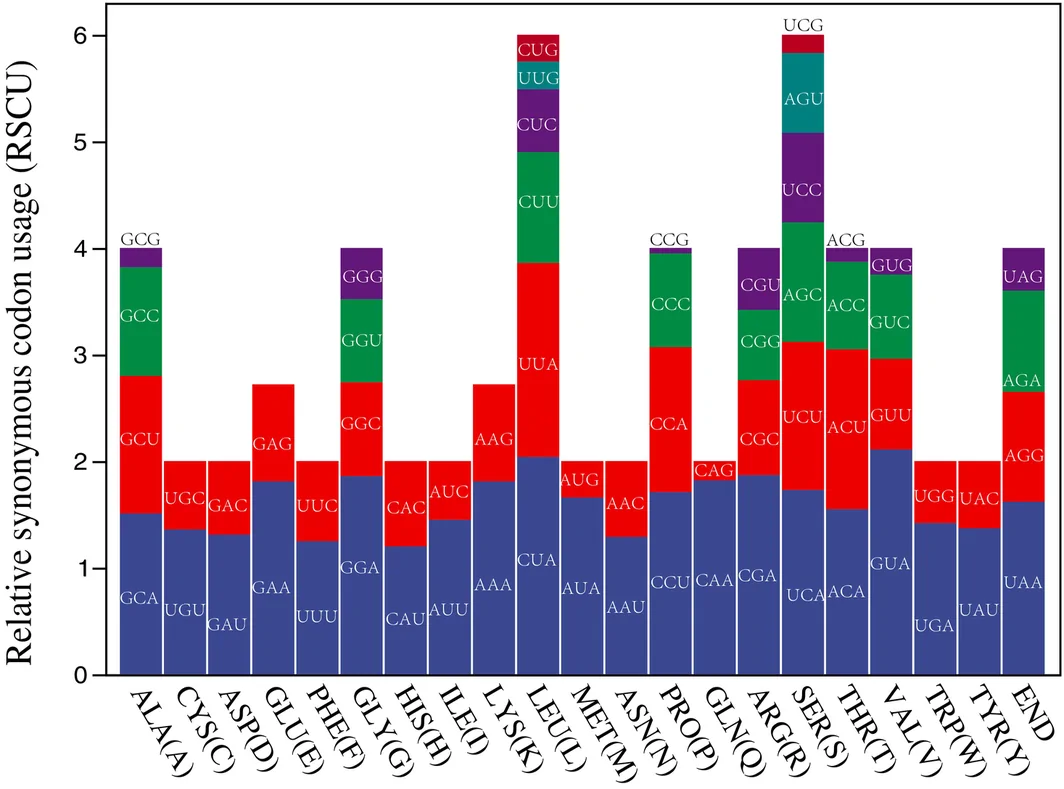
Relative synonymous codon usage (RSCU) of the PCGs of *M. chinensis*.

Analysis of codon usage patterns in *M. chinensis* revealed that the GUA codon encoding valine is the most preferred codon in this species, with a relative synonymous codon usage frequency (RSCU) of 2.11, indicating its highest usage frequency among all codons (Figure 3). Several other high‐frequency codons, including CUA (Leu, RSCU = 2.04), CGA (Arg, RSCU = 1.87), and GGA (Gly, RSCU = 1.86), also terminate with A, collectively forming the core codon usage pattern of this species. Meanwhile, some codons exhibit low usage frequency, with Pro's CCG codon (RSCU = 0.05) being the least frequently used among all codons. The codon usage patterns of *M. pequinius* and *M. chinensis* are the result of the long‐term evolution of their genomes under the combined effects of mutational pressure and possible natural selection.

**FIGURE 4:**
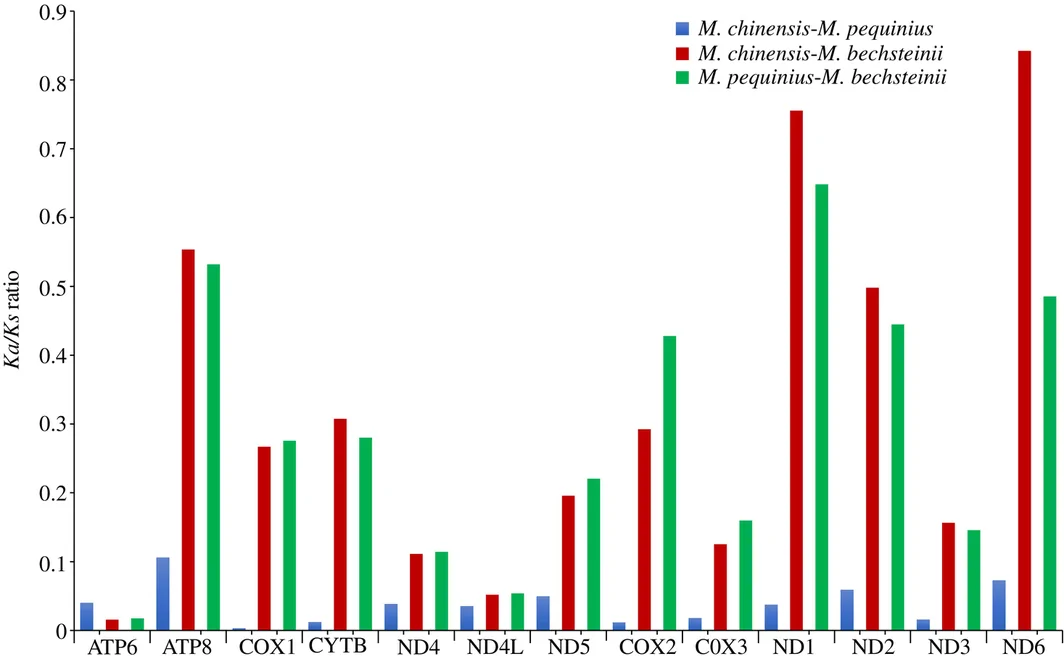
Analysis of the *Ka/Ks* for the PCGs of *M. chinensis* and *M. pequinius*.

We calculated the *Ka/Ks* ratios for all 13 PCGs across the three species. The *Ka/Ks* values for all PCGs were strictly less than 1.0 (ranging from 0.01 to 0.85, Figure 4), confirming that these genes have been predominantly shaped by purifying (negative) selection during evolutionary history. Among all PCGs, *ATP8*, *ND1*, and *ND6* exhibited relatively elevated *Ka/Ks* ratios (frequently exceeding 0.5 in pairwise comparisons).

### 3.4 rRNA、tRNA and D‐Loop

**FIGURE 5:**
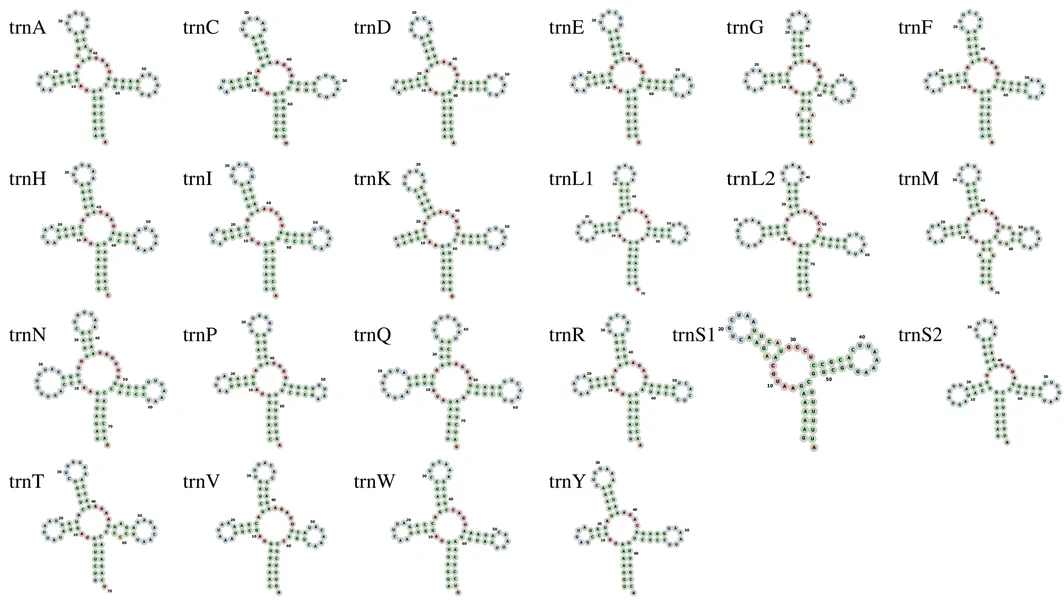
Prediction of the secondary structure of 22 tRNAs in the mitochondrial genome of *M. pequinius*.

**FIGURE 6:**
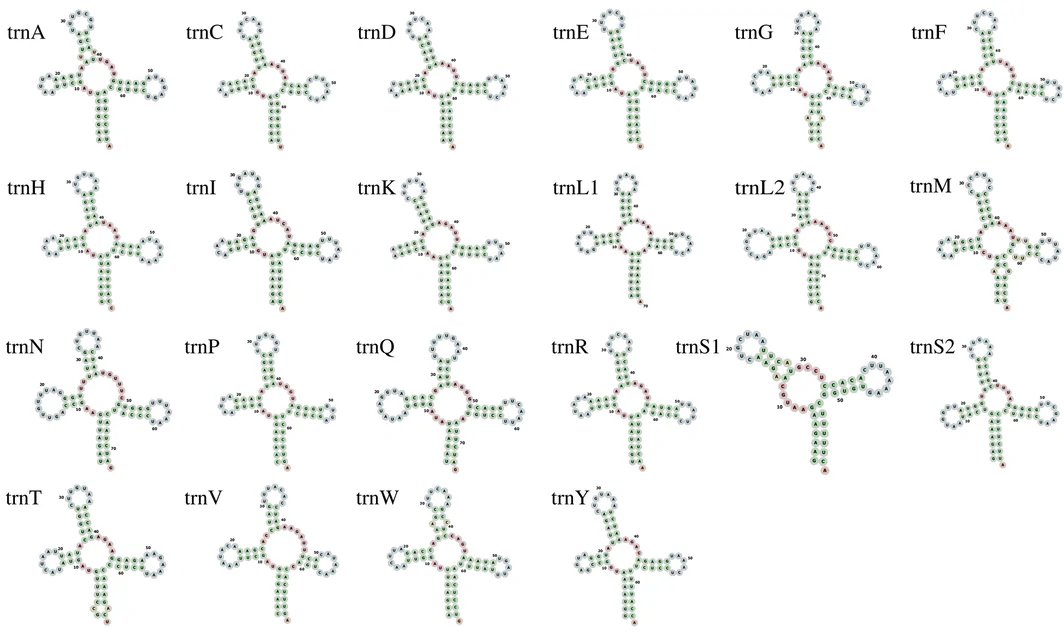
Prediction of the secondary structure of 22 tRNAs in the mitochondrial genome of *M. chinensis*.

Both the mitochondrial genome of *M. pequinius* (total tRNA length: 1509 bp) (Figure 5) and that of *M. chinensis* (total tRNA length: 1507 bp) (Figure 6) contain 22 tRNA genes, with tRNA lengths ranging from 59 bp (*trnS2*) to 75 bp (*trnL1*). Secondary structure prediction revealed that most tRNAs can fold into the standard cloverleaf structure, but the secondary structure of *trnS1* genes in both mitochondrial genomes of *M. pequinius* and *M. chinensis* was missing the DHU arm.

Both the mitochondrial genomes of *M. pequinius* and *M. chinensis* have been identified with two ribosomal RNA (rRNA) genes, namely 12S rRNA (970 bp in *M. pequinius* ; 975 bp in *M. chinensis* ) and 16S rRNA (1566 bp in *M. pequinius* ; 1562 bp in *M. chinensis* ). The 12S rRNA is located between *trnF* and *trnV*, while the 16S rRNA is situated between *trnV* and *trnL1*.

### 3.5 Phylogenetic Tree

**FIGURE 7:**
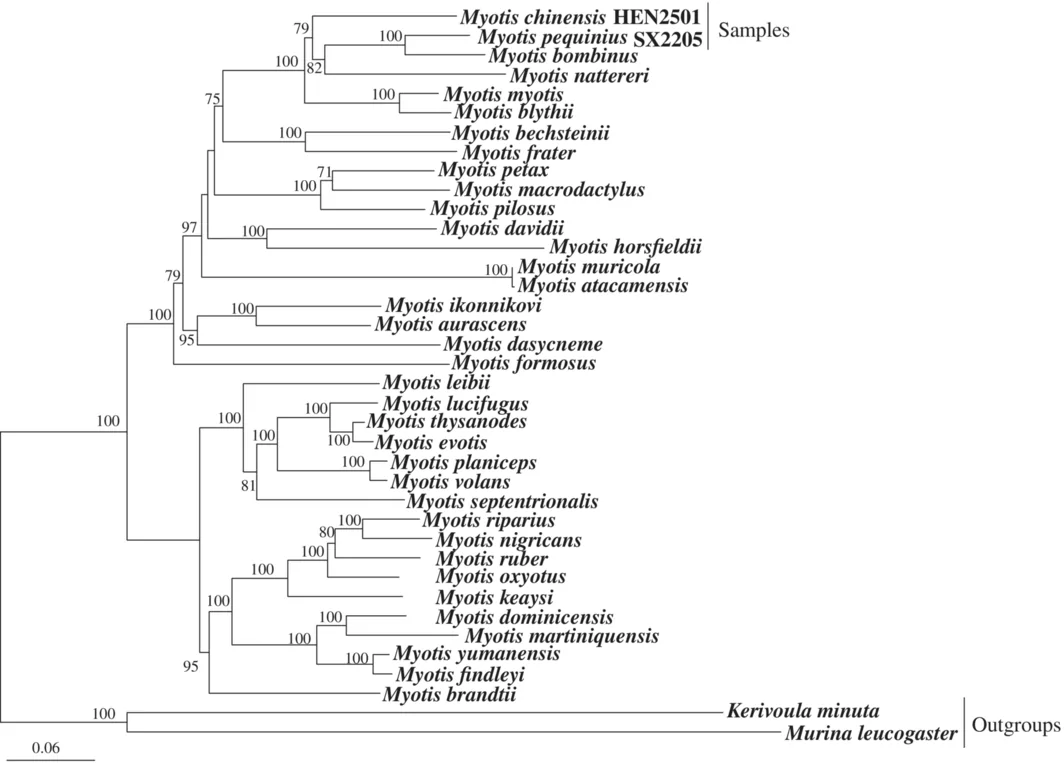
Maximum likelihood (ML) phylogenetic tree of 34 *Myotis* species based on the concatenated sequences of 13 PCGs. Numbers at nodes indicate bootstrap support values.

To clarify the phylogenetic position of *M. pequinius* (SX2205, sequence number C_AA119463.1) and *M. chinensis* (HEN2501, sequence number C_AA159671.1) within the genus *Myotis*, a maximum likelihood (ML) phylogenetic tree constructed based on 13 PCGs demonstrated (Figure 7) that all *Myotis* species form a monophyletic group, with the genus primarily divided into two major branches. *M. pequinius* and *M. bombinus* are phylogenetically closely related, clustering tightly with 100% bootstrap support, suggesting they may share a common ancestral origin. *M. pequinius* and *M. chinensis* are located in a highly supported independent clade and share a close genetic relationship. Their shared ancestral node has a support value of 79%, and they form a distinct evolutionary clade with *M. bombinus* , *M. nattereri* , *M. myotis* , and *M. blythii* and so on. This supports their unique evolutionary position within the genus *Myotis*, with a relatively distant phylogenetic relationship to other species such as *M. leibii* and *M. lucifugus* .

## 4 Discussion

In this study, we successfully assembled and annotated the mitochondrial genomes of *M. pequinius* and *M. chinensis* . The results showed that the mitochondrial genomes of the two species were similar to those of other Chiroptera species in length, gene composition, gene order, and overall structure. Both mitochondrial genomes exhibited a typical AT‐bias, and the length, structure, and coding‐strand distribution of the 13 PCGs were also largely comparable. These features are highly consistent with the mitochondrial genomic characteristics reported in other *Myotis* species (Kim et al. 2011; Nam et al. 2015; Wang et al. 2016; Yu et al. 2016; Jebb et al. 2017; Chung et al. 2018; Chen et al. 2025), reflecting the high conservation of Chiroptera mitochondrial genomes during evolution. All tRNAs in both species except *trnS1* displayed a typical cloverleaf secondary structure; the loss of the DHU arm in *trnS1* is a conserved character in metazoan mitochondrial genomes (Jühling et al. 2012; Meganathan et al. 2012; Watanabe et al. 2014).

Analysis of codon usage patterns indicated that *M. pequinius* and *M. chinensis* preferred codons ending in A or U, which is consistent with the AT‐bias of their mitochondrial genomes. However, their optimal preferred codons differed markedly: *M. pequinius* favored GGA (Gly) as the optimal codon, whereas *M. chinensis* favored GUA (Val). Such differences may be associated with species‐specific gene expression efficiency, indirectly reflecting their adaptive divergence at the molecular level. From an adaptive perspective, variation in optimal codon usage can modulate translation kinetics and protein folding efficiency, and may be linked to differential metabolic demands or ecological niches (Plotkin and Kudla 2011; Quax et al. 2015). The preferential use of GGA versus GUA in *M. pequinius* and *M. chinensis* might thus represent fine‐tuned adjustments in mitochondrial translational machinery, potentially influencing the expression of key respiratory chain components under distinct environmental pressures, though this hypothesis warrants experimental verification. In addition, the *trnS1* gene in both bat species lacks the DHU arm structure; this structural variation may represent a shared feature of the genus *Myotis*, and its effects on tRNA function and mitochondrial gene translation efficiency deserve further exploration.

Although the *Ka/Ks* ratios for all PCGs were below 1, indicating that purifying selection is the predominant evolutionary force, the ratios for *ATP8*, *ND1*, and *ND6* were relatively elevated (exceeding 0.5 in certain pairwise comparisons), suggesting that these three genes experience weaker purifying selection. *ATP8* and *ND6* are situated at peripheral or accessory positions within respiratory chain complexes V and I, respectively, and do not directly participate in core catalytic functions (Sun et al. 2017); consequently, nonsynonymous substitutions in these genes impose less detrimental impact on energy metabolism, permitting a higher tolerance for amino acid variation. Although *ND1* encodes a core transmembrane subunit of complex I, its *Ka/Ks* values were markedly increased (0.66–0.77) specifically in comparisons involving *M. bechsteini* , implying that the relaxation of selective pressure is largely confined to this lineage. This pattern may be associated with lineage‐specific environmental adaptation or demographic history—for instance, reduced effective population size, which diminishes the efficiency of purifying selection (Chen et al. 2025). Collectively, these three genes exhibit a state of reduced purifying selection rather than neutral or positive selection, and this condition may serve as a reservoir of potential variation that facilitates adaptive modulation of mitochondrial energy metabolism in response to environmental changes. It is worth noting that our Ka/Ks estimates differ somewhat from those recently reported by Chen et al. (2025) for *M. siligorensis* and *M. laniger* . In that study, all *Ka/Ks* values were below 0.12, with *ATP8* showing the highest rate (0.118), whereas we observed ratios exceeding 0.5 for *ATP8*, *ND1*, and *ND6* in certain pairwise comparisons. This discrepancy likely stems from the different species sets analyzed: our elevated ratios, particularly for *ND1*, were largely driven by comparisons involving *M. bechsteini* , suggesting a lineage‐specific relaxation rather than a genus‐wide phenomenon.

The phylogenetic tree reconstructed based on the 13 PCGs clearly divided the genus *Myotis* into two well‐supported major clades. Clade I (bootstrap = 100%) comprised the Eurasian species, including *M. bombinus* , *M. nattereri* , *M. myotis* , *M. blythii* , as well as the closely related *M. pequinius* and *M. chinensis* . Clade II (bootstrap = 100%) contained the remaining species, predominantly distributed in the Americas and East Asia. This deep bifurcation is consistent with previous molecular phylogenetic studies that have recognized these two major lineages within the genus *Myotis* based on mitochondrial and nuclear markers (Liu et al. 2023; Korstian et al. 2024). The close affinity between *M. pequinius* and *M. chinensis* within Clade I, together with their clustering with *M. bombinus* and *M. nattereri* , suggests a shared evolutionary origin among these Eurasian taxa. However, traditional morphological classifications of *Myotis* have long been plagued by convergent adaptations related to echolocation and foraging, which often obscure phylogenetic relationships and lead to conflicting taxonomic hypotheses (Liu et al. 2023). Our molecular results are largely congruent with recent phylogenetic studies (Liu et al. 2023; Korstian et al. 2024), but do not fully align with earlier morphology‐based subdivisions, emphasizing the need for integrative approaches that combine molecular, morphological and ecological data. It is important to emphasize, however, that the phylogenetic relationships inferred in this study are based exclusively on mitochondrial data. Given the well‐documented mito‐nuclear discordance within *Myotis* (Platt et al. 2018), these results should be interpreted as a mitochondrial gene tree rather than a definitive species tree. The clustering patterns observed here provide hypotheses about maternal lineage relationships that require testing with independent nuclear markers. By incorporating our newly obtained mitochondrial genomes into the existing 36 mitochondrial genome datasets of the genus *Myotis*, we provide a more comprehensive phylogenetic framework for the genus *Myotis*. Specifically, our data consolidate the placement of *M. pequinius* and *M. chinensis* within the Eurasian clade, and further resolve their sister relationship to *M. bombinus* and *M. nattereri* , a topology that was not fully supported in earlier studies with limited mitochondrial markers. This improved resolution offers a more refined mitochondrial baseline for taxonomic revisions and biogeographic inference of the genus *Myotis*, and highlights the value of complete mitochondrial genomes in refining mitochondrial lineage phylogeny.

In summary, this study reports the complete mitochondrial genomes of *M. pequinius* and *M. chinensis* for the first time, characterizes their structural features and molecular variations, and provides a mitochondrial gene tree for these species within a phylogenetic framework. Nevertheless, several limitations should be acknowledged. First, our phylogenetic and selection analyses are based exclusively on mitochondrial data, which are maternally inherited and represent a single genetic locus; nuclear genes may provide additional resolution, particularly for recent or rapid divergence events (Liu et al. 2023). Second, as extensively documented in *Myotis*, mitochondrial markers are subject to introgression, incomplete lineage sorting, and mito‐nuclear discordance, and therefore should not be used alone for definitive species‐level taxonomic inference (Puechmaille 2026; Çoraman et al. 2020; Platt et al. 2018). The phylogenetic hypotheses generated in this study require independent validation using nuclear markers. Third, the functional implications of codon usage differences and tRNA structural variations remain speculative and require experimental validation via transcriptomic or proteomic approaches. Fourth, the lineage‐specific relaxation of purifying selection observed in *ND1* warrants further investigation with larger population‐level samples to test the demographic hypothesis. Future studies incorporating population genomics—such as genome‐wide SNP data and whole‐genome resequencing—will be essential to disentangle the complex evolutionary history of the genus Myotis, to assess gene flow and introgression, and to inform conservation strategies for these ecologically important bats. These findings may facilitate phylogenetic research on the genus *Myotis* and provide essential evolutionary background information for the development of conservation strategies for related species.

## Author Contributions

**Xufan Wang:** data curation (lead), formal analysis (lead), writing – original draft (lead), writing – review and editing (lead). **Xiangxiang Cao:** investigation (supporting), visualization (supporting). **Haiyan Guan:** investigation (supporting), visualization (supporting). **Huigen Feng:** methodology (lead), validation (lead). **Sen Liu:** funding acquisition (lead), supervision (lead), writing – original draft (lead), writing – review and editing (lead).

## Conflicts of Interest

The authors declare no conflicts of interest.

## Supporting information


**Table S1:** Summary of sequencing data and assembly coverage for mitochondrial genome of the two species.

**Table S2:** Annotation and gene order in the mitochondrial genome of *Myotis pequinius* and *M. chinensis.*

## Data Availability

The mitochondrial genome sequences of *Myotis pequinius* and *M. chinensis* generated in this study are available in GenBase (National Bioinformation Center of China) under accession numbers C_AA119463.1 and C_AA159671.1, respectively. The datasets supporting the findings of this study are openly available at https://ngdc.cncb.ac.cn/genbase/. All other data are included in the manuscript and Supporting Information.
